# Antibody responses after a single dose of ChAdOx1 nCoV-19 vaccine in healthcare workers previously infected with SARS-CoV-2

**DOI:** 10.1016/j.ebiom.2021.103523

**Published:** 2021-08-12

**Authors:** Sebastian Havervall, Ulrika Marking, Nina Greilert-Norin, Henry Ng, Max Gordon, Ann-Christin Salomonsson, Cecilia Hellström, Elisa Pin, Kim Blom, Sara Mangsbo, Mia Phillipson, Jonas Klingström, Sophia Hober, Peter Nilsson, Mikael Åberg, Charlotte Thålin

**Affiliations:** a Department of Clinical Sciences, Karolinska Institutet, Danderyd Hospital, Stockholm, Sweden; b Department of Medical Cell Biology, Science for Life Laboratory, Uppsala University, Uppsala, Sweden; c Department of Protein Science, KTH Royal Institute of Technology, SciLifeLab, Stockholm, Sweden; d Public Health Agency of Sweden, Solna, Sweden; e Department of Pharmaceutical Biosciences, Science for Life Laboratory, Uppsala University, Uppsala, Sweden; f Centre for Infectious Medicine, Department of Medicine Huddinge, Karolinska Institutet, Stockholm, Sweden; g Department of Medical Sciences, Clinical Chemistry. Science for Life Laboratory, Uppsala University, Uppsala, Sweden

**Keywords:** SARS-CoV-2, Neutralizing antibody response, Prior infection, ChAdOx1 nCoV-19, BNT162b2 vaccine

## Abstract

**Background:**

Recent reports demonstrate robust serological responses to a single dose of messenger RNA (mRNA) vaccines in individuals previously infected with SARS-CoV-2. Data on immune responses following a single-dose adenovirus-vectored vaccine expressing the SARS-CoV-2 spike protein (ChAdOx1 nCoV-19) in individuals with previous SARS-CoV-2 infection are however limited, and current guidelines recommend a two-dose regimen regardless of preexisting immunity.

**Methods:**

We compared RBD-specific IgG and RBD-ACE2 blocking antibodies against SARS-CoV-2 wild type and variants of concern following two doses of the mRNA vaccine BNT162b2 in SARS-CoV-2 naïve healthcare workers (n=65) and a single dose of the adenovector vaccine ChAdOx1 nCoV-19 in 82 healthcare workers more than (n=45) and less than (n=37) 11 months post mild SARS-CoV-2 infection at time of vaccination.

**Findings:**

The post-vaccine levels of RBD-specific IgG and neutralizing antibodies against the SARS-CoV-2 wild type and variants of concern including Delta lineage 1.617.2 were similar or higher in participants receiving a single dose of ChAdOx1 nCoV-19 vaccine post SARS-CoV-2 infection (both more than and less than 11 months post infection) compared to SARS-CoV-2 naïve participants who received two doses of BNT162b2 vaccine.

**Interpretation:**

Our data support that a single dose ChAdOx1 nCoV-19 vaccine that is administered up to at least 11 months post SARS-CoV-2 infection serves as an effective immune booster. This provides a possible rationale for a single-dose vaccine regimen.

**Funding:**

A full list of funding bodies that contributed to this study can be found in the Acknowledgements section

**Research in context**

**Evidence before this study**

A PubMed search on June 21st, 2021, using search terms (“SARS-CoV-2” OR “COVID-19”) AND “vaccine” together with combinations of “natural infection”, “convalescent” and “seropositive” yielded nine publications demonstrating positive mRNA vaccine effects on immune response among individuals with previous natural infection and small or no gain of the second dose. Journal watches subscriptions and the search of reference lists added six publications to our list. We searched MedRxiv June 20th, 2021, and found an additional eleven not peer reviewed studies. Although all available evidence points towards a robust serological response to a single dose mRNA vaccine post natural infection, data on similar effects by adenovector vaccine are scarce. None of the studies we found investigated neutralizing serological responses after one dose ChAdOx1-nCoV vaccine in previously infected individuals.

**Added value of this study**

We show that a single dose of ChAdOx1 nCoV-19 vaccine to previously SARS-CoV-2 infected individuals elicits similar or higher levels of neutralizing antibodies against SARS-CoV-2 wild type and variants of concern, including Delta lineage 1.617.2, as those found following two doses of BNT162b2 vaccine in SARS-CoV-2 naïve individuals. The duration of time since natural infection did not affect the response significantly.

**Implications of all the available evidence**

Our findings support that a single dose adenovector vaccine serves as an effective immune booster after priming with natural SARS-CoV-2 infection up to at least 11 months post infection, and provides a possible rationale for a single dose vaccine regimen in individuals with prior SARS-CoV-2 infection.

Alt-text: Unlabelled box

## 1 Introduction

Recent reports demonstrate robust serological responses to a single dose of messenger RNA (mRNA) vaccines in individuals previously infected with SARS-CoV-2. These individuals develop spike-specific IgG and neutralizing antibody titers after one dose that are equivalent to or exceeding those observed after the second dose in vaccinees without preexisting immunity [1], [2], [3], [4], [5], [6], [7], [8], [9], [10]. In a study of 23 SARS-CoV-2 previously infected individuals, a substantial enhancement of neutralizing antibody response against SARS-CoV-2 variants B.1.1.7 and B.1.351 by a single dose mRNA vaccine was noted [6]. A second dose mRNA vaccine to individuals with prior SARS-CoV-2 infection add little value in terms of serological titers [1, 4, 7], and may be associated with increased risk of non-severe adverse events [11]. Data on immune responses following a single-dose adenovirus-vectored vaccine expressing the SARS-CoV-2 spike protein such as ChAdOx1 nCoV-19 in individuals with previous SARS-CoV-2 infection are however limited. Current guidelines recommend a two-dose regimen regardless of preexisting immunity [12].

We compared receptor binding domain (RBD)-specific IgG antibodies and their neutralizing capacity towards SARS-CoV-2 wild type and variants of concern, including Delta lineage 1.617.2, following two doses of the mRNA vaccine BNT162b2 in SARS-CoV-2 naïve healthcare workers and a single dose of the adenovector vaccine ChAdOx1 nCoV-19 in healthcare workers with documented SARS-CoV-2 infection up to 11 months prior to vaccination.

## 2 Methods

### 2.1 Study cohort and design

**Fig. 1:**
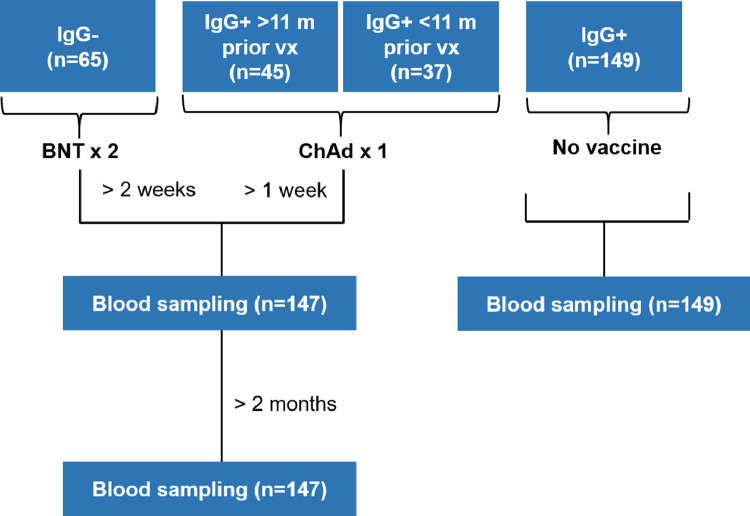
Diagram of the compilation of the study groups. BNT; BNT162b2, ChAd; ChAdOx1 nCoV-19, vx; vaccination.

The COMMUNITY (COVID-19 Biomarker and Immunity) study [13,14] investigates long-term immunity after COVID-19 in 2149 healthcare workers at Danderyd Hospital, Stockholm, Sweden, included between April 15th and May 8th, 2020. Blood samples are obtained every four months and SARS-CoV-2 spike-specific IgG are analyzed at every follow-up by multiplex antigen bead array (FlexMap3D, Luminex Corp) as previously described [13]. Between March 23rd and 31st, 2021, this sub study investigated serological responses following the second dose BNT162b2 in 65 SARS-CoV-2 naïve healthcare workers and a single dose of ChAdOx1 nCoV-19 vaccination in 82 healthcare workers with SARS-CoV-2 infection confirmed by seroconversion up to 11 months prior to vaccination (45 seroconverted > 11 months prior to vaccination and 37 seroconverted < 11 months prior to vaccination). Healthcare workers at Danderyd Hospital were offered two doses of BNT162b2 vaccine (with a 21-day interval, range 21-26 days) with start January 12th, 2021, and a first dose of ChAdOx1 nCoV-19 vaccine with start February 25th, 2021. Blood samples were obtained at least two weeks post second dose BNT162b2 and at least one week post a single dose of ChAdOx1 nCoV-19. Blood samples were also collected from all participants at least two months after vaccination. The date and type of vaccination was self-reported at study inclusion and confirmed through the Swedish regional vaccination data base VAL Vaccinera. 149 unvaccinated previously SARS-CoV-2 infected study participants (of which 61 seroconverted > 11 months prior to blood sampling and 88 seroconverted < 11 months prior to blood sampling) were enrolled as a control group. A diagram of the compilation of the study groups is presented in Figure 1, and demographics of the study groups are presented in Table 1.

**Table 1:** Demographics and levels of wild type and variants of concern B.1.1.7 (Alpha), B.1.351 (Beta), P.1 (Gamma), and B.1.617.2 (Delta) RBD-specific IgG and neutralizing antibodies 1-2 weeks post vaccination in the different study groups. Continuous variables are presented with median and interquartile range while proportions are converted to proportions that sum to 100% in the vertical axis. BNT; BNT162b2, ChAd; ChAdOx1 nCoV-19 AU; arbitrary units. kAU; kilo arbitrary units.

	BNT x 2	ChAd x 1	
	SARS-CoV-2 naïve N=65	Inf. >11 m. prior vx N=45	Inf. <11 m. prior vx N=37
Age	52 (42 - 58)	48 (41 - 59)	47 (40 - 52)
Female	57 (88%)	37 (82%)	35 (95%)
**RBD IgG (kAU/ml)**			
Wild type	49 (37 - 82)	94 (51 - 155)	56 (33 - 128)
Alpha (B.1.1.7)	49 (35 - 81)	101 (54 - 157)	58 (33 - 124)
Beta (B.1.351)	23 (16 - 36)	49 (26 - 83)	24 (12 - 67)
Gamma (P.1)	37 (25 - 56)	68 (41 - 117)	36 (19 - 89)
Delta (B.1.617.2)	52 (36 - 78)	95 (51 - 141)	55 (29 - 111)
**RBD NAb (AU/ml)**			
Wild type	46 (38 - 55)	55 (44 - 70)	53 (38 - 65)
Alpha (B.1.1.7)	41 (31 - 50)	52 (38 - 70)	45 (34 - 64)
Beta (B.1.351)	36 (21 - 47)	41 (17 - 63)	37 (21 - 58)
Gamma (P.1)	32 (24 - 35)	38 (25 - 56)	32 (25 - 47)
Delta (B.1.617.2)	41 (34 - 49)	50 (36 - 63)	43 (35 - 52)

### 2.2 Ethics

The study complied with the declaration of Helsinki, written informed consent was obtained from all participants, and the study was approved by the Swedish Ethical Review Authority (dnr 2020-01653).

### 2.3 Serological and pseudoneutralization analyses

RBD-specific IgG and their neutralizing capacity against SARS-CoV-2 wild type and variants B.1.1.7 (Alpha), B.1.351 (Beta), P.1 (Gamma), B.1.617.2 (Delta), B.1.427, B.1.525, B.1.526.2, B.1.617 and P.3 were measured using the V-PLEX SARS-CoV-2 Panel 11 for IgG and ACE2 by Meso Scale Diagnostics (MSD, USA) according to the manufacturer's instructions and expressed as arbitrary units (AU)/ml. Briefly, these assays utilize spot multiplexing and the plates were provided with in total 9 different antigens on individual spots in wells of a 96-well plate. In the serology assay, antibodies in the samples (dilution 1:50 000) bound to the antigen in the spots and a mouse monoclonal anti-human IgG conjugated with SULFO-TAG™ was used for detection. Three prediluted positive controls, corresponding to known high, medium and low antibody levels, were provided with each kit and included on every plate in duplicates. For the ACE2 neutralization assay, the ability of antibodies to block the binding of ACE2 to its cognate ligands was measured. Antibodies in samples (dilution 1:500) that block the RBD antigen interaction with ACE2 on the spots were analyzed, and a recombinant human ACE2 conjugated with MSD SULFO-TAG™ was used for detection.

All plates were analyzed on a MESO QuickPlex SQ 120 instrument, which is an electrochemiluminescence reader measuring the light emitted from the SULFO-TAG™. Results for the MSD assays are reported in AU/ml derived from back fitting the measured signals for samples to a calibration curve generated for each plate. For the 7-point calibration curve, a 4-fold serial dilution was used with two replicates at each calibrator level and an additional zero calibrator blank. By correcting for dilution, the final antibody concentrations in undiluted samples were obtained. Detailed information concerning antigens, control samples and calibrator 1, including concentrations in MSD AU/ml and WHO/NIBSC BAU/ml, are presented in the kit inserts provided by MSD (IgG Cat no. K15455U and ACE2 Cat no. K15458U).

### 2.4 Statistics

Continuous variables are presented with median and interquartile range and discrete variables are presented as proportions. For comparisons between groups, data was transformed using the log2 function as the data followed a logarithmic distribution. A regression model was generated with an interaction term for serostatus prior to vaccination and vaccination (two doses BNT162b2 vaccine or one dose ChAdOx1 nCoV-19 vaccine) with adjustment for sex and age using a dataset that included all vaccinations and pre-vaccination antibody status. Estimate comparisons are provided as multiplication factors and presented with 95% confidence intervals (CI). Due to non-normally distributed residuals, a bootstrap estimated covariance matrix was used. Analyses were performed in R version 4.1.0 with rms-package version 6.2-0.

### 2.5 Role of funding source

No funding source had role in study design, data collection, data analyses, interpretation, or writing of report.

## 3 Results

The median age of the SARS-CoV-2 naïve individuals receiving two doses BNT162b2 vaccine was 52 (42 - 58) and 88% were women. The median age of the previously SARS-CoV-2 infected individuals receiving one dose ChAdOx1 nCoV-19 vaccine > 11 months and < 11 months post SARS-CoV-2 infection was 48 (41 - 59) and 47 (40 - 52) respectively, and 82% and 95% were women, respectively. Table 1.

**Fig. 2:**
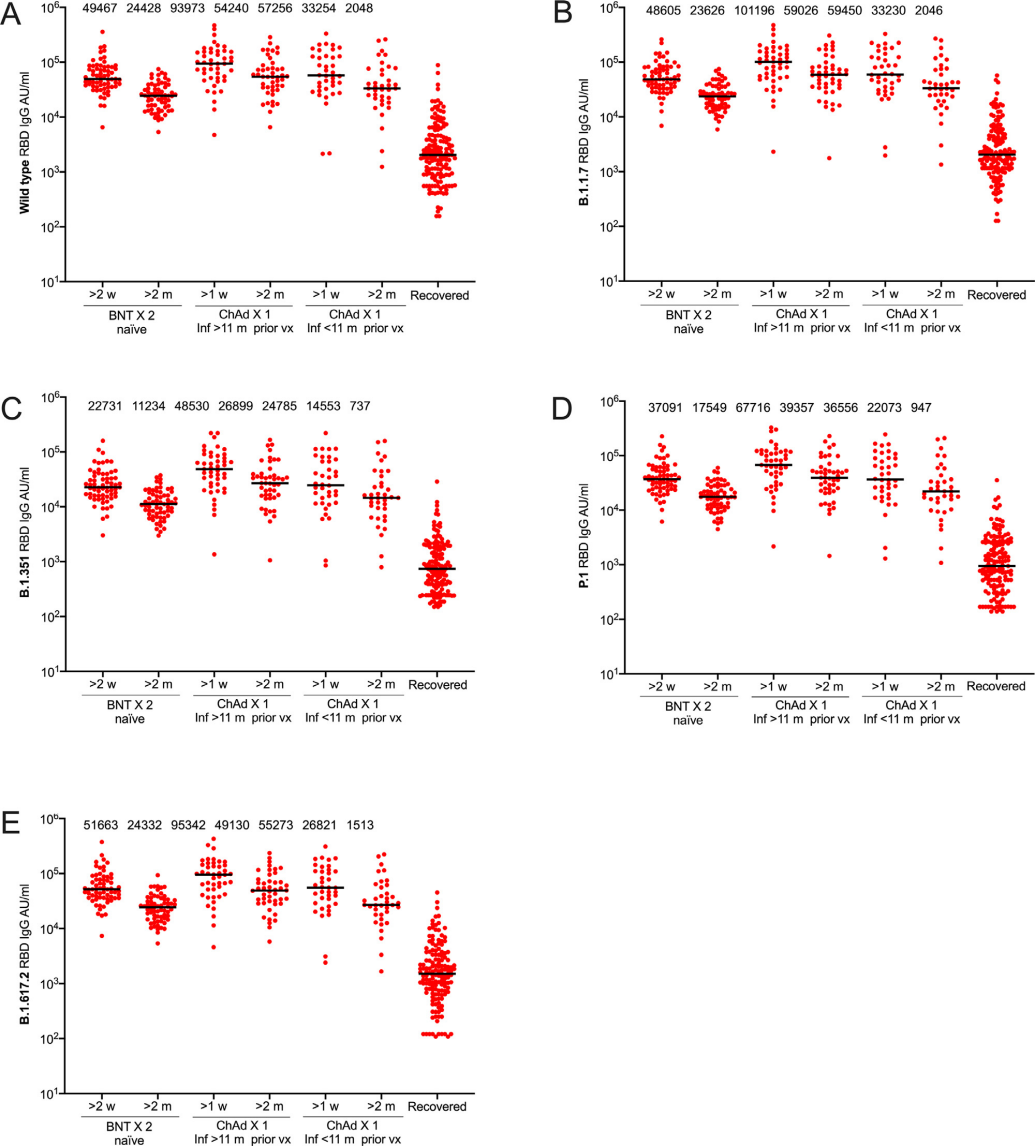
RBD-specific IgG responses against SARS-CoV-2 wild type and variants of concern following two doses BNT162b2 in SARS-CoV-2 naïve individuals or one dose ChAdOx1 nCoV-19 vaccine in individuals with previous SARS-CoV-2 infection. RBD-specific IgG responses against wild type (A), and variants of concern B.1.1.7 (B), B.1.351 (C), P.1 (D), and B.1.617.2 (E). Sample sizes: Two doses BNT162b2 in SARS-CoV-2 naïve individuals (n=65), one dose ChAdOx1 nCoV-19 > 11 mpvx (n=45), one dose ChAdOx1 nCoV-19 < 11 mpvx (n=37). Numbers above each cluster indicate median AU/ml. Lines indicate medians with interquartile ranges. BNT; BNT162b2, ChAd; ChAdOx1 nCoV-19, m; months, vx; vaccination; AU; arbitrary units.

As expected, the serological responses were higher in response to vaccine than after natural infection (Figure 2). RBD-specific IgG antibodies against the SARS-CoV-2 wild type and variants of concern B.1.1.7 (Alpha), B.1.351 (Beta), P.1 (Gamma), and B.1.617.2 (Delta) reached similar or higher levels in participants receiving a single dose ChAdOx1 nCoV-19 post SARS-CoV-2 infection (both < 11 months post infection and > 11 months post infection) than in SARS-CoV-2 naïve participants who received two doses BNT162b2 vaccine (Figure 2). The median levels of neutralizing antibodies towards the SARS-CoV-2 wild type and all four variants of concern were furthermore similar or higher in previously infected vaccinees (both < 11 months post infection and > 11 months post infection) receiving a single dose ChAdOx1 nCoV-19 compared to SARS-CoV-2 naïve participants fully vaccinated with BNT162b2 vaccine (Figure 2). Linear regression data adjusted for age and sex comparing one dose ChAdOx1 nCoV-19 post SARS-CoV-2 infection with two doses BNT162b2 in SARS-CoV-2 naïve participants are presented in Table 1. A similar trend was seen for all measured SARS-CoV-2 variants (Supplementary Table S1).

**Fig. 3:**
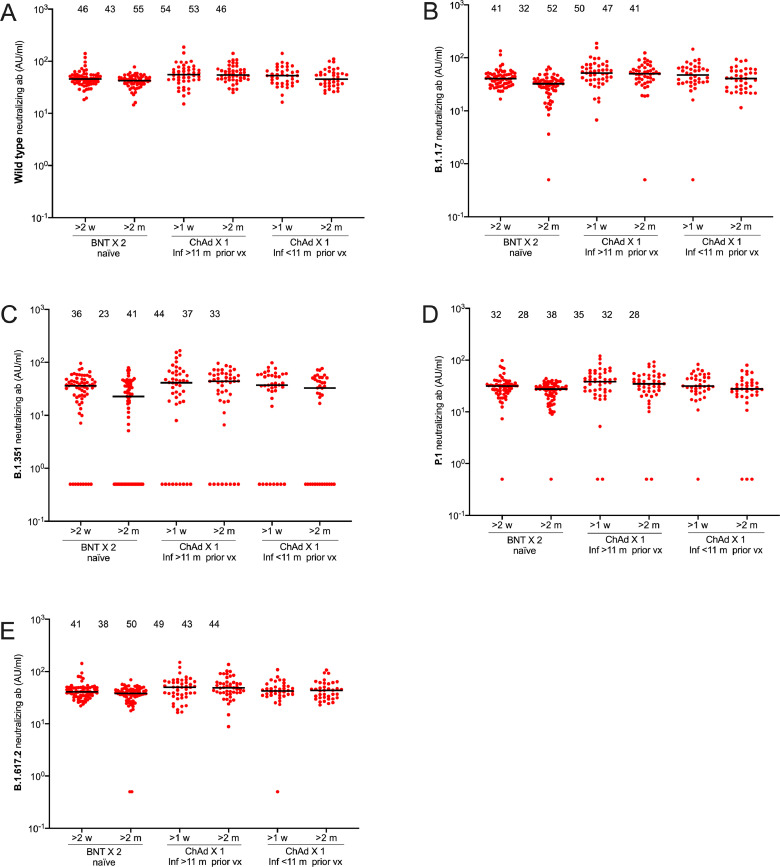
Neutralizing antibody responses against SARS-CoV-2 wild type and variants following two doses BNT162b2 in SARS-CoV-2 naïve individuals or one dose ChAdOx1 nCoV-19 vaccine in individuals with previous SARS-CoV-2 infection. Neutralizing antibody responses against wild type (A), and variants of concern B.1.1.7 (B), B.1.351 (C), P.1 (D), B.1.617.2 (E). Sample sizes: Two doses BNT162b2 in SARS-CoV-2 naïve individuals (n=65), one dose ChAdOx1 nCoV-19 > 11 mpvx (n=45), one dose ChAdOx1 nCoV-19 < 11 mpvx (n=37). Numbers above each cluster indicate median AU/ml. Lines indicate medians with interquartile ranges. BNT; BNT162b2, ChAd; ChAdOx1 nCoV-19, m;months, vx; vaccination; AU; arbitrary units.

There was an almost two-fold reduction in RBD-specific IgG antibodies against the SARS-CoV-2 wild type and variants of concern B.1.1.7 (Alpha), B.1.351 (Beta), P.1 (Gamma), and B.1.617.2 (Delta) over the first two months following vaccination (Figure 2). Notably, however, the neutralizing capacity remained relatively stable suggesting a durable immunity despite declining antibody levels (Figure 2). Both RBD-specific IgG antibodies and neutralizing antibodies remained similar or higher in vaccinees receiving a single dose ChAdOx1 nCoV-19 following infection compared to SARS-CoV-2 naïve participants fully vaccinated with BNT162b2 two months post vaccination (Figure 2, Figure 3, Table 2, Table 3). A similar trend was seen for all SARS-CoV-2 variants two months post vaccination (Supplementary Table S2).

**Table 2:** Estimate comparisons of one dose ChAdOx1 nCoV-19 in participants with prior SARS-CoV-2 infection > 1 week post vaccination with two doses BNT162b2 in SARS-CoV-2 naïve participants > 2 weeks post vaccination in the reference group. The estimate provides the multiplication factor for how much the AU/ml is modified by switching from two doses BNT162b2 in SARS-CoV-2 naïve participants to a single ChAdOx1 nCoV-19 dose in participants with prior SARS-CoV-2 infection. Estimates around 1.0 suggest no difference. All values are adjusted for sex and age. Inf; infection, m; months, vx; vaccination, RBD; receptor binding protein, NAb; neutralizing antibodies. CI; confidence interval.

	RBD IgG				RBD NAb	
	Estimate	95% CI			Estimate	95% CI
Wild type						
Inf <11m prior vx	1.0	(0.7 to 1.4)			1.1	(0.9 to 1.3)
Inf >11m prior vx	1.6	(1.2 to 2.1)			1.2	(1.0 to 1.4)
Alpha (B.1.1.7)						
Inf <11m prior vx	1.1	(0.7 to 1.5)			1.0	(0.7 to 1.3)
Inf >11m prior vx	1.6	(1.2 to 2.2)			1.2	(1.0 to 1.5)
Beta (B.1.351)						
Inf <11m prior vx	1.0	(0.6 to 1.4)			0.7	(0.3 to 1.8)
Inf >11m prior vx	1.8	(1.2 to 2.5)			0.9	(0.4 to 2.2)
Gamma (P.1)						
Inf <11m prior vx	0.9	(0.6 to 1.4)			1.0	(0.6 to 1.6)
Inf >11m prior vx	1.6	(1.1 to 2.2)			1.0	(0.5 to 1.7)
Delta (B.1.617.2)						
Inf <11m prior vx	0.9	(0.6 to 1.3)			0.9	(0.5 to 1.1)
Inf >11m prior vx	1.4	(1.0 to 1.9)			1.1	(0.9 to 1.3)

**Table 3:** Estimate comparisons of one dose of ChAdOx1 in participants with prior SARS-CoV-2 infection >2 months post vaccination with two doses BNT162b2 in SARS-CoV-2 naïve participants >2 months post vaccination in the reference group. The estimate provides the multiplication factor for how much the AU/ml is modified by switching from two doses BNT162b2 in SARS-CoV-2 naïve participants to a single dose ChAdOx1 nCoV-19 in participants with prior SARS-CoV-2 infection. Estimates around 1.0 suggest no difference. All values are adjusted for sex and age. Inf; Infection, m; months, vx; vaccination, RBD; receptor binding protein, NAb; neutralizing antibodies. CI; confidence interval.

	RBD IgG				RBD NAb	
	Estimate	95% CI			Estimate	95% CI
Wild type						
Inf <11m prior vx	1.4	(1.0 to 2.1)			1.2	(1.0 to 1.3)
Inf >11m prior vx	2.3	(1.7 to 3.1)			1.4	(1.2 to 1.6)
Alpha (B.1.1.7)						
Inf <11m prior vx	1.5	(1.0 to 2.2)			1.5	(1.2 to 1.9)
Inf >11m prior vx	2.4	(1.7 to 3.2)			1.7	(1.1 to 2.5)
Beta (B.1.351)						
Inf <11m prior vx	1.4	(0.9 to 2.0)			1.1	(0.4 to 3.6)
Inf >11m prior vx	2.4	(1.7 to 3.2)			3.6	(1.3 to 9.4)
Gamma (P.1)						
Inf <11m prior vx	1.3	(0.9 to 1.9)			0.8	(0.4 to 1.4)
Inf >11m prior vx	2.2	(1.6 to 3.0)			1.2	(0.8 to 1.9)
Delta (B.1.617.2)						
Inf <11m prior vx	1.2	(0.9 to 1.8)			1.1	(1.0 to 1.2)
Inf >11m prior vx	2.0	(1.5 to 2.7)			1.2	(1.0 to 1.4)

## 4 Discussion

Recent data suggest that the first mRNA vaccine dose induces equal to or stronger immune responses in individuals with prior SARS-CoV-2 infection as those following the second mRNA vaccine dose in SARS-CoV-2 naïve individuals. These emerging studies challenge current vaccine guidelines recommending a two-dose regimen regardless of preexisting immunity [15]. Data on a single adenovector vaccine following natural infection is, however, scarce. Our findings show that also a single dose of the adenovector vaccine ChAdOx1 nCoV-19 following natural infection elicits a robust and durable serological response with broad neutralizing capacity against SARS-CoV-2 wild type and variants of concern including Delta lineage 1.617.2, and that these immune responses exceed those after two doses of the mRNA vaccine BNT162b2 in SARS-CoV-2 naïve individuals.

Notably, the strong serological response of a single dose ChAdOx1 nCoV-19 was found both after less than eleven months and more than eleven months post infection. This suggests that a single dose adenovector vaccine is sufficient to induce an effective immune response in individuals that have been exposed to the SARS-CoV-2 virus as long as up to a year prior to the time of vaccination. The participants in this study were furthermore not hospitalized during or following the SARS-CoV-2 infection, indicating that also a mild disease is sufficient as a priming of the immune response prior a single vaccine dose.

The study is limited by the small sample size and observational and single-center nature. The study cohort moreover comprised a majority of women, but age-wise reasonably representative of the working population in Sweden.

Taken together, our data support that a single dose adenovector vaccine serves as an effective immune booster after priming with natural SARS-CoV-2 infection up to at least 11 months post infection, with immune responses against SARS-CoV-2 wild type and variants of concern exceeding those found after two doses mRNA vaccine in SARS-CoV-2 naïve individuals. These findings propose that a single dose adenovector vaccine is sufficient to provide the immune boost needed to achieve high levels of immunity in this population. Noteworthy, however, there may be practical challenges in identifying individuals with prior SARS-CoV-2 infection, and antibody screening prior to vaccination is both costly and time consuming in the midst of a pandemic. Nevertheless, our data provides a rationale for a single dose vaccine regimen in individuals with prior SARS-CoV-2 infection, but further studies will need to confirm whether the results are generalizable to the wider population.

## Contributors

SeH, UM, MÅ, and CT designed the study, made statistical analysis, did the figures, and drafted the manuscript. NG, HN, ACS, CH, EP, MÅ conducted the analysis and the administrative and material support. SeH, UM, KB, SM, JK, MP, MÅ, SoH, PN, CT interpreted the data, and all authors critically revised the manuscript. MG made statistical analyses. SoH, PN, CT obtained funding. MP, SoH, PN, CT supervised. SeH and CT has verified the underlying data.

## Declaration of Competing Interest

SoH has participated on Astra Zeneca COVID-19 SCG Virtual Advisory Board. Otherwise, the authors declare no competing interests.

## Appendix Supplementary materials

Image, application 1
