# Supplementary material for: Antibody responses after a single dose of ChAdOx1 nCoV-19 vaccine in healthcare workers previously infected with SARS-CoV-2
Source: eBioMedicine. 2021 Aug 12;70:103523. doi: 10.1016/j.ebiom.2021.103523 (PMC8357428; doi:10.1016/j.ebiom.2021.103523)
Supplement: Supplementary file 1 [file mmc1.docx]

**Supplementary data**

Table S1. RBD-specific IgG and neutralizing antibodies against SARS-CoV-2 wild type and variants in the different study groups > one week post vaccination. Continuous variables are presented with median and interquartile range while proportions are converted to proportions that sum to 100% in the vertical axis. BNT; BNT162b2, ChAd; ChAdOx1 nCoV-19, vx; vaccination, RBD; receptor binding domain, IQR; interquartile range, AB; antibodies. AU; arbitrary units. kAU; kilo arbitrary units.

|  | BNT x 2 |  |  | ChAd x 1 | |
| --- | --- | --- | --- | --- | --- |
|  | N=65 |  |  | Inf. >11 m. prior vx N=45 | Inf. <11 m. prior vx N=37 |
| **RBD IgG (kAU/ml)** | | | | | |
| Wildtype | 49 (37 - 82) |  |  | 94 (51 - 155) | 56 (33 - 128) |
| B.1.1.7 (Alpha) | 49 (35 - 81) |  |  | 101 (54 - 157) | 58 (33 - 124) |
| B.1.351 (Beta) | 23 (16 - 36) |  |  | 49 (26 - 83) | 24 (12 - 67) |
| B.1.427 | 59 (44 - 90) |  |  | 111 (61 - 169) | 63 (33 - 133) |
| B.1.525 | 36 (24 - 57) |  |  | 69 (35 - 121) | 37 (20 - 95) |
| B.1.526.2 | 47 (34 - 70) |  |  | 81 (47 - 133) | 50 (28 - 107) |
| B.1.617 | 54 (41 - 91) |  |  | 103 (56 - 165) | 58 (29 - 128) |
| B.1.617.2 (Delta) | 52 (36 - 78) |  |  | 95 (51 - 141) | 55 (29 - 111) |
| P.1 (Gamma) | 37 (25 - 56) |  |  | 68 (41 - 117) | 36 (19 - 89) |
| P.3 | 22 (15 - 36) |  |  | 48 (25 - 81) | 26 (14 - 70) |
| **RBD NAb (AU/ml)** | | | | | |
| Wildtype | 46 (38 - 55) |  |  | 55 (44 - 70) | 53 (38 - 65) |
| B.1.1.7 (Alpha) | 41 (31 - 50) |  |  | 52 (38 - 70) | 45 (34 - 64) |
| B.1.351 (Beta) | 36 (21 - 47) |  |  | 41 (17 - 63) | 37 (21 - 58) |
| B.1.427 | 41 (35 - 49) |  |  | 47 (33 - 66) | 44 (34 - 55) |
| B.1.525 | 49 (40 - 58) |  |  | 53 (39 - 67) | 52 (33 - 69) |
| B.1.526.2 | 43 (36 - 53) |  |  | 54 (36 - 67) | 45 (37 - 59) |
| B.1.617 | 39 (32 - 46) |  |  | 44 (34 - 59) | 38 (30 - 55) |
| B.1.617.2 (Delta) | 41 (34 - 49) |  |  | 50 (36 - 63) | 43 (35 - 52) |
| P.1 (Gamma) | 32 (24 - 35) |  |  | 38 (25 - 56) | 32 (25 - 47) |
| P.3 | 36 (27 - 46) |  |  | 38 (28 - 57) | 39 (26 - 56) |
|  | | | | | |

Table S2. RBD-specific IgG and neutralizing antibodies against SARS-CoV-2 wild type and variants in the different study groups > one week post vaccination. Continuous variables are presented with median and interquartile range while proportions are converted to proportions that sum to 100% in the vertical axis. BNT; BNT162b2, ChAd; ChAdOx1 nCoV-19, vx; vaccination, RBD; receptor binding domain, IQR; interquartile range, AB; antibodies. AU; arbitrary units. kAU; kilo arbitrary units.

|  | BNT x 1 |  |  | ChAd x 1 | |
| --- | --- | --- | --- | --- | --- |
|  | N=65 |  |  | Inf. >11 m. prior vx N=45 | Inf. <11 m. prior vx N=37 |
| **RBD IgG (kAU/ml)** | | | | | |
| Wildtype | 24 (15 - 35) |  |  | 54 (35 - 78) | 33 (22 - 72) |
| B.1.1.7 (Alpha) | 24 (16 - 36) |  |  | 60 (35 - 83) | 33 (24 - 72) |
| B.1.351 (Beta) | 11 (8 - 17) |  |  | 27 (16 - 39) | 15 (9 - 27) |
| B.1.427 | 28 (18 - 38) |  |  | 61 (34 - 88) | 33 (22 - 68) |
| B.1.525 | 18 (11 - 25) |  |  | 37 (22 - 57) | 21 (13 - 32) |
| B.1.526.2 | 22 (14 - 32) |  |  | 48 (30 - 68) | 28 (20 - 62) |
| B.1.617 | 27 (18 - 37) |  |  | 57 (32 - 83) | 30 (18 - 56) |
| B.1.617.2 (Delta) | 24 (15 - 34) |  |  | 50 (29 - 73) | 27 (18 - 56) |
| P.1 (Gamma) | 18 (12 - 27) |  |  | 39 (23 - 55) | 22 (14 - 37) |
| P.3 | 11 (8 - 16) |  |  | 30 (15 - 40) | 15 (9 - 25) |
| **RBD NAb (AU/ml)** | | | | | |
| Wildtype | 43 (37 - 49) |  |  | 53 (45 - 69) | 46 (36 - 60) |
| B.1.1.7 (Alpha) | 32 (24 - 38) |  |  | 50 (35 - 61) | 41 (27 - 57) |
| B.1.351 (Beta) | 23 (0.09 - 39) |  |  | 44 (18 - 60) | 33 (0.09 - 50) |
| B.1.427 | 36 (30 - 40) |  |  | 44 (35 - 57) | 38 (31 - 47) |
| B.1.525 | 38 (31 - 45) |  |  | 47 (39 - 61) | 38 (28 - 55) |
| B.1.526.2 | 38 (32 - 45) |  |  | 49 (39 - 62) | 44 (31 - 58) |
| B.1.617 | 41 (34 - 47) |  |  | 48 (40 - 61) | 42 (33 - 51) |
| B.1.617.2 (Delta) | 38 (33 - 43) |  |  | 44 (37 - 55) | 42 (35 - 48) |
| P.1 (Gamma) | 28 (20 - 32) |  |  | 35 (25 - 49) | 28 (21 - 36) |
| P.3 | 35 (25 - 40) |  |  | 48 (40 - 56) | 37 (25 - 52) |
|  | | | | | |
